# Atypical Motor-Predominant Presentation of Subacute Combined Degeneration of Spinal Cord Due to Vitamin B12 Deficiency: A Case Report of Stroke Mimicry in the Absence of Classic Symptoms

**DOI:** 10.7759/cureus.74697

**Published:** 2024-11-28

**Authors:** Zara Saeed, Syeda Ariba Zehra, Anwar Muhammad, Imran Ashraf

**Affiliations:** 1 Medicine, Dartford and Gravesham NHS Trust, London, GBR; 2 Internal Medicine, Dartford and Gravesham NHS Trust, London, GBR; 3 Stroke Medicine, Dartford and Gravesham NHS Trust, London, GBR

**Keywords:** gait disturbance, neurological manifestations, stroke, subacute combined degeneration, vitamin b12 deficiency

## Abstract

Vitamin B12 deficiency is a prevalent condition that can lead to serious neurological disorders, including subacute combined degeneration (SCD) of the spinal cord, which can result in lasting damage if not promptly treated. This report discusses a unique case of a 53-year-old female patient who presented with a one-week history of gait instability and falls, ultimately diagnosed with SCD due to severe vitamin B12 deficiency. Notably, the patient exhibited an atypical presentation, lacking classic symptoms such as paraesthesia and hematologic abnormalities, which often accompany B12 deficiency.

Clinical evaluation revealed no previous neurological deficits or hematological issues, suggesting an acute decline in B12 levels. Neuroimaging, particularly MRI of the spine, showed demyelination affecting the dorsal columns and corticospinal tracts, consistent with SCD while excluding ischemic causes. Laboratory tests confirmed low serum B12 levels, supporting the diagnosis.

This case underscores the necessity for clinicians to maintain a high index of suspicion for vitamin B12 deficiency in patients presenting with unexplained neurological symptoms. Early intervention is crucial; in this case, immediate treatment with intramuscular B12 led to significant recovery in gait stability and neurological function. The findings highlight the importance of thorough assessments and ongoing education about the diverse presentations of B12 deficiency, especially in populations at risk, such as the elderly and those with malabsorption issues. Awareness of the neurological consequences of B12 deficiency is essential for effective management and improved patient outcomes.

## Introduction

Vitamin B12 deficiency is typically associated with hematologic abnormalities, particularly macrocytic anemia, but its effects extend beyond the blood to the nervous system [1]. Neurological manifestations of B12 deficiency can be subtle and varied, often presenting as nonspecific symptoms such as paraesthesia, gait disturbances, and cognitive changes. In more severe and advanced cases, deficiency can lead to subacute combined degeneration (SCD) of the spinal cord, a potentially irreversible condition characterized by demyelination of the dorsal and lateral columns of the spinal cord. This condition is most commonly associated with pernicious anemia but can also occur in individuals with dietary B12 deficiency, especially in those following strict vegetarian or vegan diets without supplementation [2].

Subacute combined degeneration primarily affects the dorsal columns, which are responsible for proprioception and fine touch, and the lateral columns, which transmit motor and pain sensations. The loss of myelin in these areas leads to a gradual loss of motor function, balance disturbances, and sensory deficits. Patients may experience ataxia, muscle weakness, and impaired deep tendon reflexes. Advanced cases can result in significant disability, including paralysis and sensory loss. The hallmark features of SCD are progressive ataxia, weakness, and paresthesia, especially in the lower limbs [3]. Neurological deficits often progress stepwise, leading to worsening gait disturbances, clumsiness, and, in severe cases, paralysis.

The pathophysiology of SCD is rooted in the disruption of myelin synthesis due to inadequate levels of vitamin B12, a crucial cofactor in the methylation cycle involved in myelin production. Vitamin B12 deficiency leads to the accumulation of methylmalonic acid and homocysteine, both of which are neurotoxic and contribute to demyelination. The dorsal columns in the spinal cord are most vulnerable due to their high demand for myelin and reliance on B12-dependent biochemical processes. If left untreated, the demyelination can cause irreversible neurological damage.

Given the potential for permanent neurological impairment, early diagnosis and intervention are paramount [4]. Vitamin B12 replacement therapy can reverse the hematologic abnormalities and halt the progression of neurological deficits if initiated promptly. This case underscores the necessity for a high index of suspicion for vitamin B12 deficiency in patients presenting with sudden-onset neurological symptoms, particularly in the absence of typical systemic signs such as anemia. Even in the absence of overt hematologic signs, neurological symptoms such as gait abnormalities and sensory disturbances should prompt consideration of vitamin B12 deficiency as a potential cause [5].

## Case presentation

A 53-year-old female with no significant medical history presented to the hospital with a one-week history of reduced mobility and recurrent falls. She did not report pain, paresthesia, or sensory loss in the lower limbs. The patient indicated that she had been a vegetarian for several years and took multivitamins regularly.

**Figure 1:**
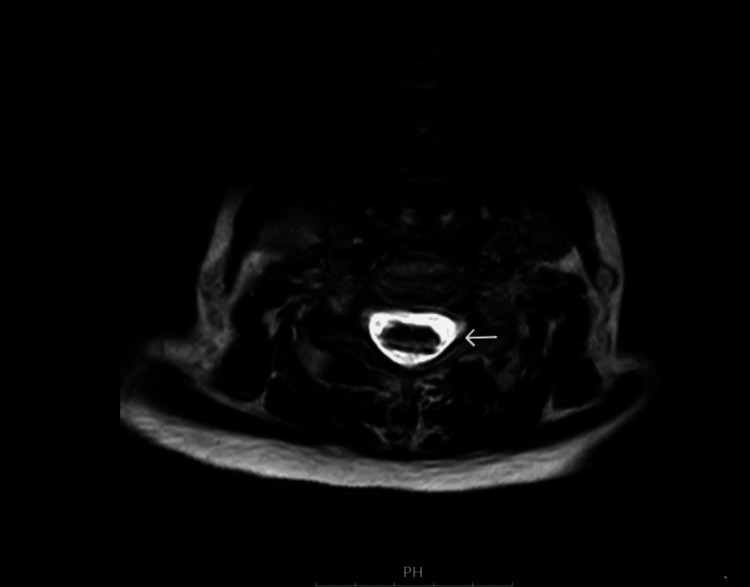
Axial view of the T2 sequence of MRI spine shows hyperintensity

**Figure 2:**
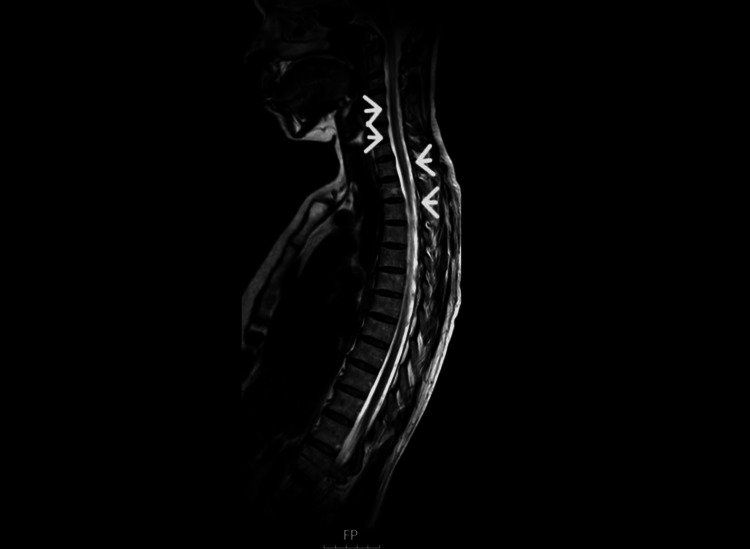
Sagittal view of the TW2 sequence of the MRI spine shows an increased signal in the dorsal and lateral columns of the cervical and thoracic region TW2: Tanner-Whitehouse 2

Physical examination revealed a wide-based gait and reduced mobility without muscle weakness or sensory deficits. The cranial nerve examination was normal. No optic atrophy was noted on fundoscopy. Motor examination revealed a power of 5/5 bilaterally in both limbs, normal tone, normal superficial and deep sensations, and brisk lower limb reflexes. Ataxia was noted on the heel-shin test. Initial laboratory investigations showed macrocytic red blood cells and a significantly low serum B12 level (<50 ng/L). The coeliac screen and anti-intrinsic factor antibodies were negative. Thus, the diagnosis of dietary vitamin B12 deficiency was made. Given her acute neurological presentation, an initial differential diagnosis of stroke was considered, and a non-contrast CT head scan was performed, which revealed no hemorrhage. An MRI of the brain showed no ischemic changes; however, an MRI of the spine revealed subacute demyelination in the dorsal columns, consistent with SCD secondary to vitamin B12 deficiency (Figures 1-2).

The patient was started on vitamin B12 replacement immediately, with an initial loading dose of 1 mg every other day for two weeks, which resulted in a dramatic improvement in her symptoms. Afterward, she was discharged home following an assessment by physiotherapists, who deemed her safe for discharge with support from the community neurological rehabilitation team. She was subsequently reviewed in the neurology clinic one month after discharge. At that visit, she reported no falls since her discharge and noted an improvement in her mobility. She is now able to mobilize independently indoors but uses a trolley outdoors due to a lack of confidence and fear of falling. She continues to be regularly followed up by consultants in both the falls and stroke clinics. Currently, she receives vitamin B12 injections once a month and is also being consistently monitored by the community neurological rehabilitation team.

## Discussion

This case represents a unique presentation of SCD, where the primary symptoms were gait disturbances and falls, without classic features such as sensory loss, paresthesia, or pronounced hematologic abnormalities beyond mild macrocytosis. Unlike other case reports, which often cite progressive weakness, paresthesia, and neuropsychiatric symptoms, this patient's presentation was primarily motor-based, mimicking stroke, a diagnostic consideration due to her acute symptom onset [6,7]. The neurological impairments associated with vitamin B12 deficiency have been documented in varying frequencies, with one study finding that approximately 40% of vitamin B12-deficient patients present with neuropsychiatric symptoms even in the absence of anemia [8]. 

In the differential diagnosis of patients with neurological symptoms and macrocytic anemia, vitamin B12 deficiency should be considered, particularly given its potential reversibility with prompt treatment. Studies show that early treatment with vitamin B12 can lead to a significant recovery in neurological function, with improvement rates of up to 80% within the first year of therapy [9]. This case contributes to the literature by underscoring the need for clinicians to consider vitamin B12 deficiency as a differential diagnosis in patients with atypical neurological presentations, as highlighted in a similar case report [10].

## Conclusions

This case highlights a rare presentation of SCD due to vitamin B12 deficiency, marked by a stroke-like onset of gait instability and falls rather than the more typical sensory symptoms or hematological abnormalities. Subacute combined degeneration, which results from vitamin B12 deficiency, primarily affects the spinal cord and peripheral nervous system, leading to demyelination. In this patient, the neurological symptoms initially mimicked a stroke, making the diagnosis challenging.

This case underscores the importance of maintaining a high index of suspicion for vitamin B12 deficiency in patients presenting with sudden neurological symptoms, especially when macrocytosis is present, even without clear signs of anemia. Early recognition and prompt treatment with vitamin B12 supplementation can significantly improve outcomes, as demonstrated here, where the patient experienced substantial recovery in gait and balance following treatment. Clinicians should remain vigilant when assessing patients with unexplained neurological symptoms, particularly those at risk for B12 deficiency, such as older adults, individuals with gastrointestinal disorders, or those on restrictive diets. Timely intervention can prevent further neurological damage and enhance recovery.
